# Impact of Different Crystalloids on the Blood Glucose Levels of Nondiabetic Patients Undergoing Major Elective Surgeries

**DOI:** 10.7759/cureus.34294

**Published:** 2023-01-27

**Authors:** Rahul Kurra, Ravi Madhusudhana

**Affiliations:** 1 Anesthesiology, Sri Devaraj Urs Medical College, Kolar, IND

**Keywords:** hyperglycemia, perioperative, ringer's lactate, dextrose normal saline, potassium chloride

## Abstract

Background

This study aimed to compare intraoperative blood sugar level fluctuations between a group of patients receiving Ringer's lactate (RL) fluid as maintenance fluid and another group receiving 0.45% dextrose normal saline with 20 mmol/liter potassium.

Materials and methods

This randomized double-blind study was conducted on 68 nondiabetic patients undergoing elective major surgeries at R. Laxminarayanappa Jalappa Hospital, Sri Devaraj Urs Medical College, Kolar, during the academic year from Jan 2021 to May 2022. Informed consent was obtained from these patients concerning their participation in this study. There were two groups of patients: Ringer lactate (RL) was administered to group A, and 0.45% dextrose normal saline and 20 mmol/L potassium chloride (KCl) were administered to group B. The vitals and blood glucose levels were measured among the patients. A p-value of 0.05 was considered statistically important.

Results

The mean age of the patients was found to be 43.60 ± 15 years, with comparable age and gender distribution between the groups. On comparison of the mean blood glucose levels immediately after induction was not important between the groups. The mean levels were comparable between the groups (p>0.05). After completion of the surgery, the mean blood glucose level significantly increased in group B patients when compared to those in group A (p<0.05).

Conclusion

The study found a substantial increase in intraoperative blood glucose levels among patients receiving 0.45% dextrose normal saline with 20 mmol/liter potassium instead of RL solution as maintenance fluid.

## Introduction

Maintaining a circulating plasma volume ensures organ perfusion and oxygen delivery to tissues, which is the aim of perioperative fluid treatment [1]. Increased catabolic activity results from an increase in the production of catabolic hormones such as cortisol and glucagon. Neoglucogenesis and hyperglycemia are elevated because of these endocrine and metabolic alterations; hence, the prevalence of hyperglycemia can be used to evaluate this stress response [2].

Insulin treatment has been shown to reduce endothelial activity, preserve hepatic mitochondria, increase glucose absorption, increase circulatory lipid levels and decrease inflammatory markers [3]. The hyperglycemic response may be influenced by the kind of anesthesia used during surgery. It has been shown that as compared to local and epidural analgesia, general anesthesia causes higher blood glucose concentrations [4,5].

Intraoperative glucose management is essential for enhancing surgical results, even in nondiabetic patients. Tight glycemic control has an economic benefit because it has been shown to improve patient outcomes related to the length of stay, stroke, renal failure, and mortality.

In this context, we examined how different maintenance fluids affected levels of glucose in nondiabetic persons following major surgery.

## Materials and methods

After obtaining the Institutional Ethics Committee's approval (SDUMC/KLR/609/2020-21 dated 24/12/2020), a randomized double-blind study was designed. A total of 68 nondiabetic patients undergoing major elective surgeries at R. Laxminarayanappa Jalappa Hospital, Kolar, India, were enrolled as study subjects. Informed consent was obtained from all of the study participants.

The inclusion criteria for this study were patients aged 20 to 60 years, both genders, an American Society of Anaesthesiologists physical status 1 or 2, and patients who would undergo major elective surgery. The duration of recruitment was from Jan 2021 to May 2022.

Patients with uncontrolled glycaemic profiles, uncompensated systemic illness (hepato-renal, cardiovascular, and endocrine), alcohol/substance abuse issues, smokers and morbid obesity, and serum potassium levels >5 Meq/l were excluded from this study.

Randomization was conducted based on computerized randomization codes, and two groups were formed.

Sampling procedure 

Detailed clinical histories of the patients were obtained, and routine investigations were checked. Once the patient was shifted to OT, their capillary blood glucose (CBG) level was noted, and monitoring was initiated.

The patients were pre-medicated with 0.2mg glycopyrrolate, 1mg midazolam, pantoprazole 40mg, and ondansetron 4mg IV before induction of anesthesia. Induction was carried out with propofol 2mg per kilogram, and tracheal intubation was facilitated by succinylcholine 1mg per kg. Anaesthesia was maintained with 66% N2O and sevoflurane in 33% O2 on controlled ventilation.

The patients were separated into two groups: group A who received Ringer's lactate (RL), and group B who received 0.45% dextrose with normal saline and KCL 20 mmol/L.

The labels of the IV fluids of each patient were wrapped by an OT staff member and given to the anesthetist who attended the surgery to perform the study, and the person who conducted this study did not get to know which fluid was given either. Thus, double blinding was achieved. Based on their body weight and overnight fasting, the hourly infusion rate was calculated, which was balanced out by the maintenance fluid using the 4:2:1 method. Mops and a suction bottle were used to roughly determine Segar's blood loss and the patients' plasma loss.

Additionally, the patients' comfort levels were observed. CBG was assessed following surgery.

Statistical analysis

The sample size was calculated based on a study by Khetarpal et al. [6]. The collected data were coded and entered into an Excel database (Microsoft, Redmond, Washington). All of the qualitative/categorical measures, such as gender and the American Society of Anesthesiologists (ASA) physical status, were summarised as frequency and percentage and analyzed using the Chi-squared test. Independent sample t-test, Mann-Whitney U-test, and a Chi-squared test/Fisher's exact test were considered appropriate to interpret the results. Quantitative variables such as weight, blood pressure, and heart rate were summarised as mean and standard deviation and analyzed using unpaired Student's t-test. A p-value of <0.05 was considered as statistically significant and analyzed using SPSS v21 operating on Windows 10 (IBM Inc., Armonk, New York).

## Results

In the present study, a total of 68 participants were included after obtaining their informed consent. The patients were then randomly distributed into the following two groups: group A who received RL, and group B who received 0.45% dextrose normal saline and potassium chloride 20 mmol/L.

The mean age of the patients was found to be 43.60±15.3 years, with a minimum of 20 and maximum age of 70 years (Table 1).

**Table 1:** Patients' mean age

	N	Minimum	Maximum	Mean	SD
Age	68	20	70	43.60	15.30

The mean ages between the groups were comparable, with no significant differences between them (p>0.05), as shown in Table 2.

**Table 2:** Mean age comparison

	Group A		Group B		p-value
	Mean	SD	Mean	SD	
Age	45.43	14.91	42.15	15.61	0.385

On the assessment of the vitals between the groups, it was found that there was comparable heart rate, blood pressure, mean arterial pressure, and baseline blood glucose between the group's preoperative periods (Table 3). 

**Table 3:** Comparison of the vitals and baseline blood glucose between the groups PR - pulse rate, SBP - systolic blood pressure, DBP - diastolic blood pressure, MAP - mean arterial pressure, CBG - capillary blood glucose

	Group A		Group B		p-value
	Mean	SD	Mean	SD	
Pre-op PR	88.1	8.7	92.0	10.9	0.110
Pre-op SBP	125	8	124	8	0.557
Pre-op DBP	79.9	8.7	80.3	7.9	0.835
Pre-op MAP	94.0	7.5	94.3	7.1	0.857
Baseline CBG	98.0	4.6	95.6	7.0	0.08

On comparing the mean blood glucose levels immediately after induction, it was found to be not significant between the groups. Moreover, the mean levels were comparable between the groups (p>0.05), as shown in Table 4.

**Table 4:** Comparison of change in CBG between the groups immediately after induction CBG - capillary blood glucose

	Group A		Group B		p-value
	Mean	SD	Mean	SD	
Immediate after induction CBG	98.4	3.5	99.4	6.8	0.474

After completion of the surgery, the mean blood glucose level was significantly increased in the patients in group B compared to those in group A (p<0.05), as shown in Table 5.

**Table 5:** Comparison of change in CBG between the groups after the completion of surgery CBG - capillary blood glucose

	Group A		Group B		p-value
	Mean	SD	Mean	SD	
After completion of surgery CBG	100.3	5.0	120.5	12.1	0.001*

## Discussion

Even in nondiabetic persons, intraoperative glycemic management plays an essential role in enhancing surgical outcomes. Perioperative hyperglycemia has a complicated etiology: physiologic stress increases sympathetic activation, which raises levels of glucagon, catecholamines, growth hormone, and cortisol [7-9].

An increase in endogenous glucose synthesis by gluconeogenesis (mainly hepatic) and glycogenolysis is brought on by the rise in counter-regulatory hormones. Under typical physiological circumstances, skeletal, cardiac, adipose (GLUT4), and liver (GLUT2) insulin-mediated glucose absorption in peripheral tissues, together with a reduction in hepatic glucose production, carefully maintains glucose homeostasis. [10,11].

The current evidence-based practice recommends that intraoperative fluid management be tailored for two key therapeutic contexts: in low-risk patients undergoing low-risk or ambulatory surgery, high-volume crystalloid infusions of 20-30 ml kilogram improve ambulatory anesthetic outcomes, including pain, nausea, dizziness, and preparedness for the street. However, a 'restrictive' hydration regimen appears to be beneficial for high-risk individuals undergoing major surgery. Patients' risk of developing post-operative infections will increase as a result of intraoperative hyperglycemia. In comparison to patients without the condition, those with intraoperative hyperglycemia had a higher rate of operative site infection. The majority of nondiabetic individuals undergoing moderate- to high-risk surgery experienced intraoperative hyperglycemia. Therefore, the risk factors for intraoperative hyperglycemia must be determined [12].

In this study, intraoperative blood glucose level changes were compared between patients receiving RL as a maintenance fluid and a control group receiving 0.45% dextrose NaCl. The mean age of the patients was found to be 43.60±15.3 years, with a minimum of 20 and maximum age of 70 years. The patients were randomly assigned to one of two groups: Group A received RL, while Group B was given 0.45% dextrose normal saline with potassium chloride. The mean ages and gender distributions among the groups were comparable.

As in the case of the present study, Kaur et al. documented no significant difference in mean age between the groups, and comparable gender distribution was also noted [13]. In another study by Chin et al., the mean age of patients was 38 years, with equal gender distribution [14].

Mean arterial pressure, HR, blood pressure, and baseline plasma glucose levels were comparable when the vital signs were compared between the groups. Additionally, the mean blood sugar levels between the groups did not significantly differ right after induction. The mean levels were similar amongst the groups (p>0.05). Following surgery, group B patients' mean blood glucose levels were significantly higher than those of group A patients (p<0.05).

Chin et al. discovered a considerable difference in plasma glucose levels across groups an hour after infusion, even though 33% of patients receiving dextrose saline had plasma glucose levels of 8 mmol/l. However, nondiabetic persons can also experience considerable, albeit brief, hyperglycemia after a small 500 ml dose [14].

According to Maitra et al., only 63% of patients in group B (RL) had at least one episode of hyperglycemia, compared to 29% in group A (0.45% sodium chloride with 5% dextrose). Group B ingested much more insulin than group A to maintain normoglycemia. The relative risk of getting hyperglycemic in group B individuals was 2.172. In group B, the number necessary to induce injury, i.e., hyperglycemia, was 2.941. Stress-induced hyperglycemia is prevalent in nondiabetic persons after major non-cardiac surgery, and the risk increases when RL fluid is substituted for maintenance fluid treatment. Much greater doses of human normal insulin are needed in patients receiving dextrose-containing saline as maintenance fluid to achieve normoglycemia with intravenous bolus [15].

Despite the low-calorie load in a study by Chin et al., 500 mL of 5% dextrose in 0.9% normal saline resulted in severe hyperglycemia. However, this was only temporary, and its clinical importance is unknown. Nonetheless, acute hyperglycemia has been linked to paired phagocytosis by polymorphonuclear leukocytes, complement system malfunction, and increased sympathoadrenergic activity. Furthermore, perioperative hyperglycemia has also been linked to glycosuria, longer hospital admissions, more frequent wound infections, ischemic episodes, and lower survival over two years in the context of coronary artery bypass graft surgery.

RL solution can be used as an alternative in nondiabetic persons undergoing major surgery and is most likely a different IV fluid for perioperative care. Within the first few hours after surgery and throughout the intraoperative period, CBG levels in group 2 rose considerably (p<0.001). According to the current study, patients who received 0.45% Dextrose NaCl with 20 mmol/L potassium as their maintenance fluid had considerably higher intraoperative blood glucose levels than patients who received RL fluid as their maintenance fluid. Researchers discovered that patients who utilized RL as maintenance fluids had a lower incidence of hyperglycemia.

## Conclusions

It is surmised that stress-induced hyperglycemic response is common in nondiabetic persons undergoing major surgery. According to the current study, patients who received 0.45% dextrose normal saline with 20 mmol/liter potassium as their maintenance fluid instead of RL solution had considerably higher intraoperative blood glucose levels. In patients receiving dextrose-containing saline as maintenance fluid, soluble human regular insulin was required to achieve normal CBG levels by IV bolus dose. Intraoperative glucose management is essential for enhancing surgical results, even in nondiabetic patients. Tight glycemic control has an economic benefit because it has been shown to improve patient results such as length of stay, stroke, renal failure, and mortality. The study found that patients who used RL as maintenance fluid had a decreased incidence of hyperglycemia.
